# Early Callus as an Adverse Prognostic Sign Following Radial Head Replacement for Trauma

**DOI:** 10.7759/cureus.72813

**Published:** 2024-11-01

**Authors:** Helen Nuttall, Angelos Assiotis

**Affiliations:** 1 Trauma and Orthopaedics, Lister Hospital, Stevenage, GBR

**Keywords:** aseptic loosening, elbow trauma, radial head arthroplasty, radial head fracture, radial head replacement

## Abstract

Radial head fractures are common elbow injuries. Radial head replacement (RHR) is a well-established surgical option for non-reconstructible fractures, either in isolation or alongside associated elbow trauma, such as a terrible triad injury, which is a complex injury characterized by elbow dislocation and coronoid and radial head fractures. The procedure aims to restore the stabilizing function of the radial head, which plays a key role in maintaining longitudinal and valgus stability and an auxiliary role in the varus stability of the elbow. Radial head arthroplasty has gained popularity in recent years, especially with the introduction of anatomically designed prostheses, though it is not without complications. We present a case series of four patients with an early post-operative radiographic sign of callus arising from the lateral aspect of the radial neck at the resection level. This sign should alert treating clinicians that there may be undetected fracture line propagation to the radial neck and a high risk of early prosthetic loosening. Based on our series, consideration of the causes behind the aseptic loosening of press-fit radial head replacement (RHR) implants should include undetected fracture lines of the radial neck.

## Introduction

Radial head fractures are common injuries, amounting to around 30% of elbow fractures and up to 5% of all fractures overall [1]. Restoration of the anatomy of the radial head is important, as the radio-capitellar joint accommodates around 60% of the longitudinal load that goes through the elbow [2]. The radial head also serves as a secondary stabilizer against valgus loading and assists the medial collateral ligament in this function. Finally, it also has a role to play in varus stability, as the native radial head applies appropriate tension to the lateral collateral ligament complex and allows it to function as a stabilizer against varus loading [3].

In the context of trauma, radial head replacement (RHR) is a surgical intervention primarily indicated for non-reconstructible radial head fractures [4]. Several designs have been used to replace the radial head and there has been an evolution from non-anatomical and non-modular implants, to anatomical and modular implants that allow accurate restoration of native anatomy. Furthermore, RHR implants may be cemented or uncemented. Some uncemented designs are inserted as press-fit implants, where initial rigid fixation to the host bone is required in order for bone growth to occur. This initial implant stability after insertion is crucial for this fixation method to be successful and long-lasting.

Despite implant design advancements and updates in surgical approaches and techniques, postoperative complications remain a significant concern. One of these complications is RHR aseptic loosening, which often results in the need for revision surgery [5]. We present a case series of four patients who all presented with early callus lateral to the resection level on the radial neck after RHR and who had either early loosening after their operations or an identified fracture line extending to the radial neck during the index procedure. This early post-operative radiographic sign should be identified and when present, should alert clinicians to the higher risk of early loosening of the implant.

## Case presentation

In the context of monitoring surgical outcomes in our unit, we carried out an audit in our patient cohort who had RHRs for trauma for the four years between 2020 and 2023. Within this timeframe, we performed 63 RHRs for trauma. Out of this cohort of patients, we identified four patients, two males and two females, who underwent RHR for radial head fractures and who presented with a callus on the lateral aspect of the radial neck stump, soon after surgery. In one of these patients, an extension of the fracture line into the radial metaphysis was noted during the index operation and she had a long-stemmed implant inserted due to this observation. Although an early callus appeared at the radial neck soon after surgery, her implant has remained stable. The other three patients underwent standard RHRs and in all three, an early callus was noted in the lateral aspect of the radial neck, and subsequent early loosening of their implants was diagnosed. In all four cases presented in our series, the implant used was the Acumed Anatomic Radial Head (Acumed, Hillsboro, USA). This system is a modular monopolar, press-fit implant with a titanium stem and a cobalt-chromium head that has several sizes and combinations of the head, neck, and stem diameters, allowing for the recreation of native radial head anatomy. In all cases, the radial head was replaced through an extensor digitorum communis splitting approach. Out of a total of 63 patients with RHRs, there were no further cases of aseptic loosening noted other than the cases presented in this series, at the point of reviewing their radiographs.

Case 1

**Figure 1:**
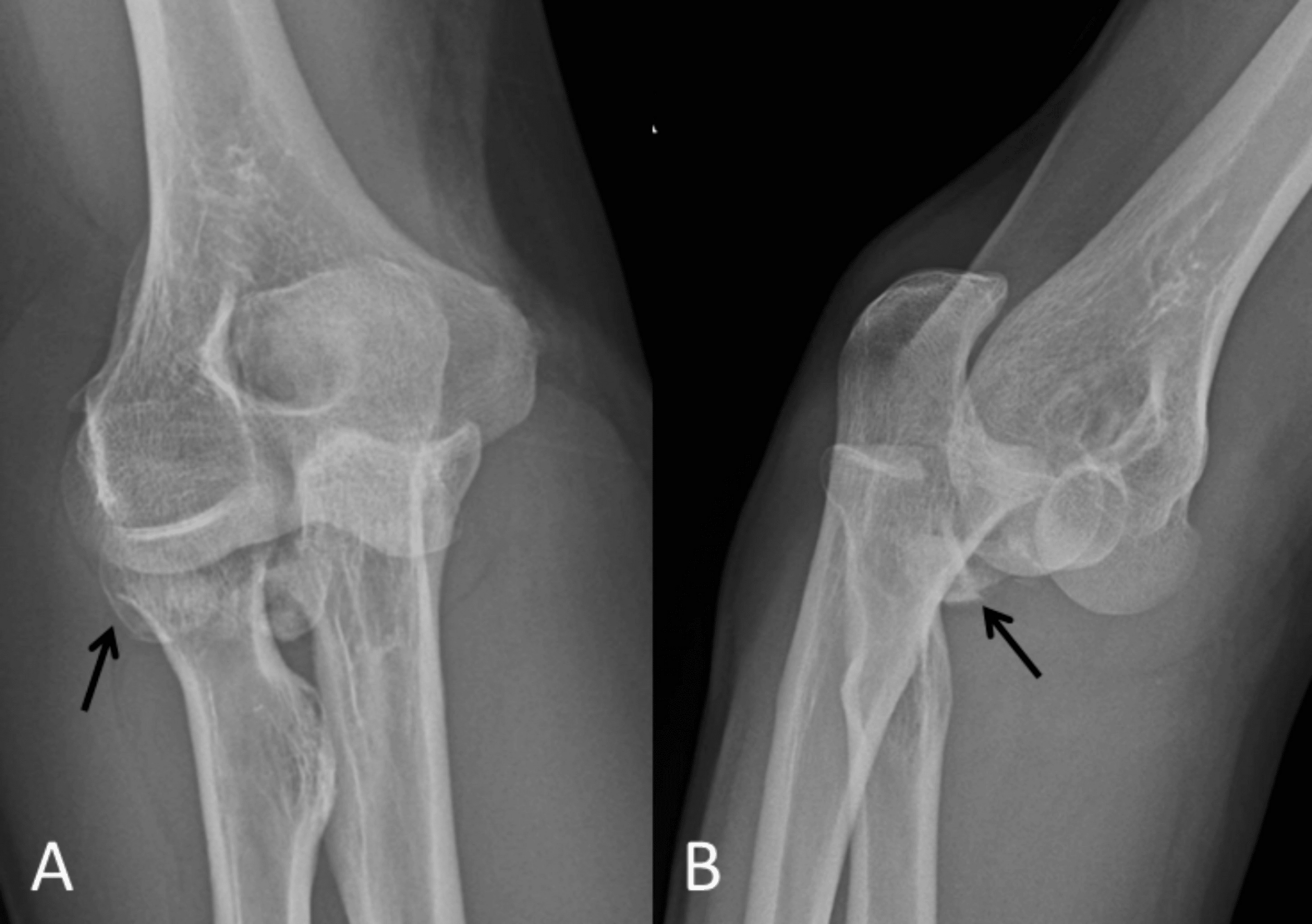
Pre-operative radiographs. A: Antero-posterior view demonstrating a highly comminuted and displaced fracture of the radial head (radial head fragments demonstrated by black arrow). B: Lateral views demonstrating the associated elbow dislocation and one of the displaced radial head fragments (black arrow).

A 62-year-old left-handed male patient fell off his bicycle at moderate speed and landed heavily on his right upper limb. He sustained a fracture and dislocation of the elbow, with a radial head comminuted fracture, a fracture of the tip of the coronoid process, and an injury to the lateral collateral ligament of the elbow. His elbow was successfully reduced in the Emergency Department (ED) and three days later he was operated on (Figure 1).

During his surgery, the fragments of his radial head were excised, as it was deemed to be non-reconstructible, and an appropriately sized RHR was inserted. His lateral ulnar collateral ligament was repaired with an all-suture anchor to the lateral epicondyle and his coronoid tip fragment was stabilized with transosseous sutures through the ulna. No untoward events were noted in the operating theatre and his elbow was stable across a normal range of movement at the end of the procedure. Rehabilitation started on the following day with an early range of movement exercises.

**Figure 2:**
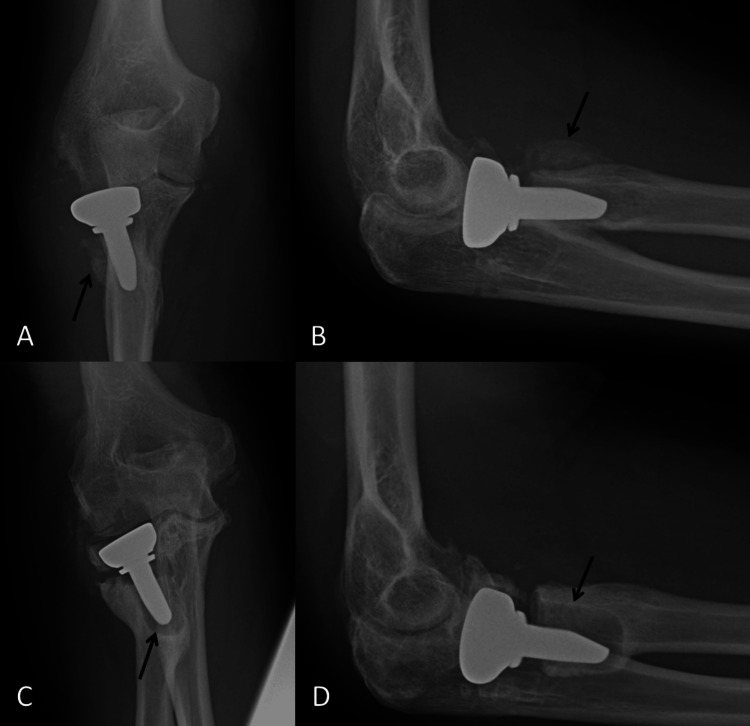
Post-operative radiographs at four weeks and 12 months after surgery. A: Antero-posterior view at four weeks after surgery demonstrating callus formation at the resection level of the radial neck (black arrow). B: Lateral view at four weeks after surgery demonstrating the same callus formation as image A (black arrow). C: Antero-posterior view at twelve months after surgery demonstrating significant loosening of the implant (black arrow). D: Lateral view at 12 months after surgery demonstrating significant loosening of the implant (black arrow).

His first post-operative radiograph took place four weeks after surgery and this demonstrated significant callus formation to the lateral border of the radial neck, at the level of the resection. He was initially asymptomatic, so we continued to monitor him at regular intervals. At 12 months after his index operation, he presented with lateral elbow pain and radiographs demonstrated that the RHR was loose. At 14 months after the index operation, the RHR was removed and his pain has improved. His range of movement is from 5° to 130° in the flexion-extension axis and he has complete supination and pronation of the forearm (Figure 2).

Case 2

**Figure 3:**
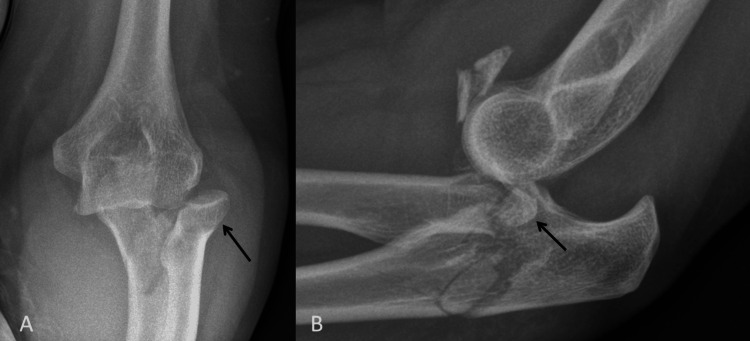
Pre-operative radiographs demonstrating a fracture/dislocation of the elbow. A: Antero-posterior view showing dislocated radial head (black arrow), which is also fractured. B: Lateral view demonstrating significant comminution of radial head (back arrow) and also, a comminuted fracture of the ulna.

A 55-year-old right-handed female patient fell off a ladder and landed on her left elbow. She presented to our ED with a fracture and dislocation of the elbow, which included a comminuted radial head fracture and a comminuted proximal ulna fracture with involvement of the coronoid process. After an initial reduction in the ED, she was operated on the same day (Figure 3).

During her operation, her radial head was replaced and her proximal ulna was stabilized with an olecranon locking plate. Her lateral ulnar collateral ligament was also repaired with an anchor to the lateral epicondyle. At the end of the procedure, she had a stable elbow across the entire range of movement of the joint. Rehabilitation started on the following day with early range of movement exercises.

**Figure 4:**
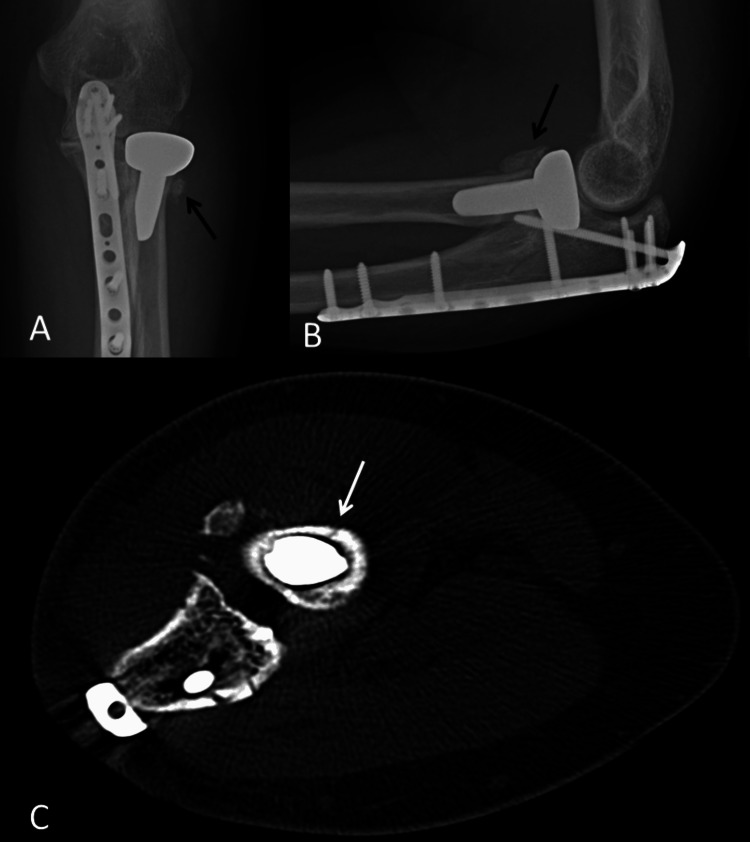
Post-operative radiograph and CT scan. A: Antero-posterior view at four weeks after surgery demonstrating callus formation lateral to the radial neck (black arrow). B: Lateral view at four weeks after surgery demonstrating callus formation (black arrow) and a reduced elbow joint. C: CT scan slice taken eight weeks after surgery, demonstrating an undisplaced fracture line through the radial neck at the level of the radial RHR (white arrow). RHR, radial head replacement

Her first post-operative radiograph took place four weeks after surgery and it was noted that she had callus formation at the implant-bone interface at the level of the radial neck. At that time, she complained of mild pain around her proximal radius. Blood tests noted a normal white cell count and C-reactive protein and clinically there was no evidence of infection. As the pain persisted, she had a CT scan eight weeks after surgery, which demonstrated the callus around the radial neck but also showed a fracture line in the radial metaphysis, at the level of the stem of the RHR. Her range of movement at 15 months after surgery is 30°-100° in the flexion-extension arc, 30° of pronation, and 20° of supination. She has been placed on our unit’s waiting list and is awaiting revision or excision of the RHR and an open elbow arthrolysis (Figure 4).

Case 3

**Figure 5:**
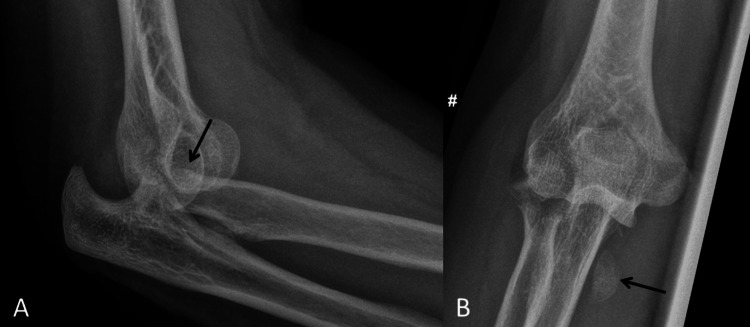
Pre-operative radiographs demonstrating a fracture/dislocation of the elbow. A: Lateral view demonstrating a fracture of the radial head, with the radial neck appearing truncated (black arrow). B: Antero-posterior view demonstrating the severe comminution of the radial head and a radial head fragment displaced medial to the proximal ulna (black arrow).

A 69-year-old right-handed female patient fell while walking outdoors, landing on the right upper limb. She sustained a fracture and dislocation of the right elbow. Her radial head was comminuted with significant displacement of fragments. One of the radial head fragments was noted to be over the medial aspect of the ulna. She also had evidence of a coronoid tip fracture. Her elbow was reduced in the ED and she had surgery two days after her presentation (Figure 5).

In the operating theatre, the radial head fragments were excised as the fracture was deemed to be non-reconstructible. She had a suture repair of the coronoid process fragment with transosseous sutures through the ulna, an anchor repair of the lateral ulnar collateral ligament, and then attention was turned to the radial head replacement (RHR). It was noted then that the fracture line extended into the proximal radial metaphysis and that the lateral aspect of the radial neck had presented with an unstable cortical fragment. The decision was made to implant a long-stemmed press-fit RHR in order to ensure implant stability.

**Figure 6:**
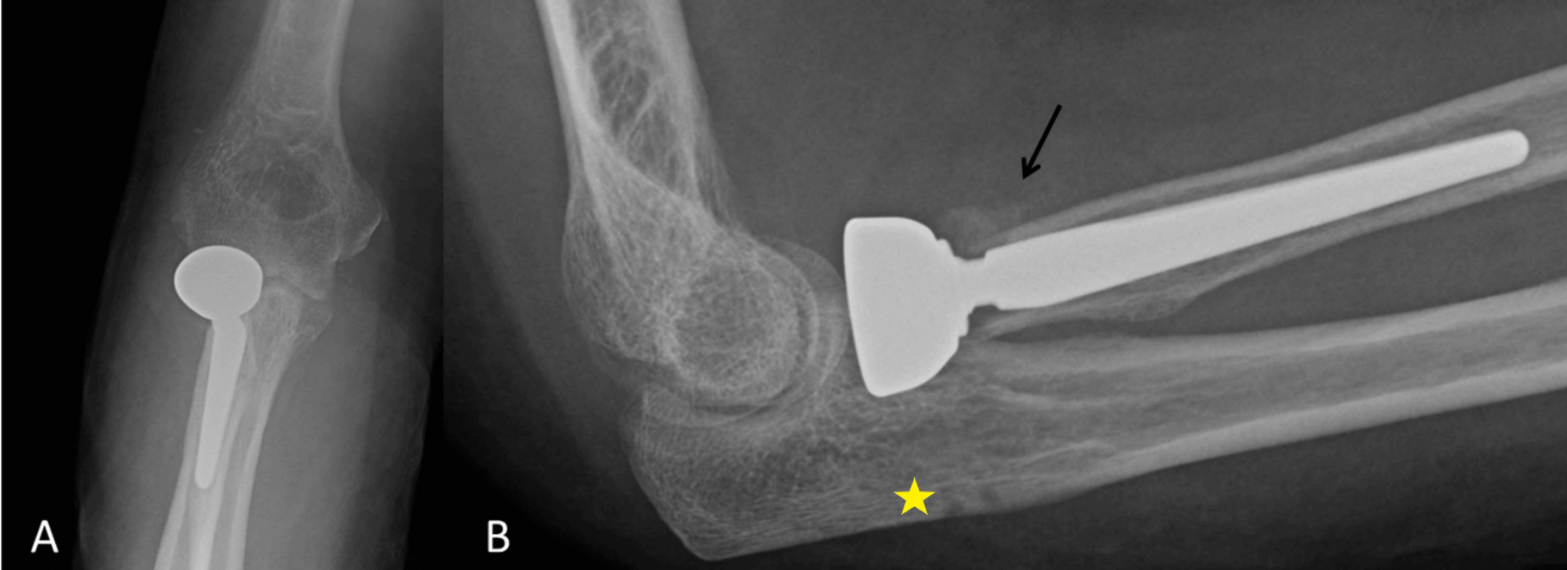
Post-operative radiographs at four weeks after surgery. A: Antero-posterior view demonstrating a well-fitting long-stemmed RHR. B: Lateral view demonstrating the appearance of callus in the lateral radial neck (black arrow), at the site of the fracture that was noted intra-operatively. Also, note the transosseous drill holes in the ulna that were used to repair the coronoid tip fracture (yellow asterisk). RHR, radial head replacement

Her first post-operative radiograph took place in the clinic four weeks after surgery, and a callus over the lateral aspect of the radial neck was noted. This did not have any negative impact on her recovery and at 14 months after surgery, she has full pronation and supination, and her flexion-extension range of movement is 10°-130° (Figure 6).

Case 4

**Figure 7:**
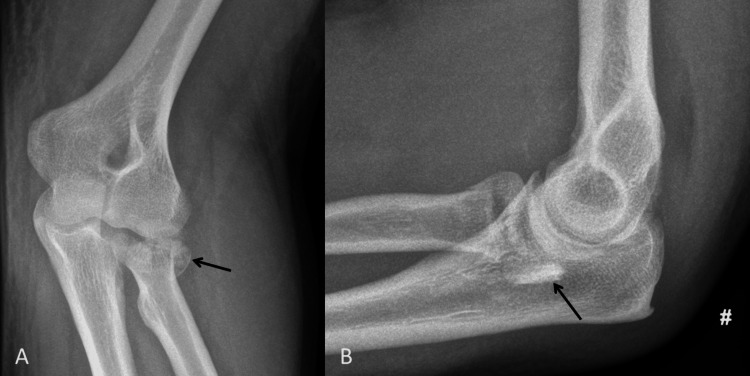
Pre-operative radiographs. A: Antero-posterior view demonstrating a highly comminuted and displaced fracture of the radial head (black arrow). B: Lateral view demonstrating a large displaced fragment of the radial head articular surface (black arrow).

A 44-year-old right-handed male patient slipped while walking on a wet floor and landed on his left hand. He presented to the ED with a highly comminuted and displaced radial head fracture, with around 50% of the articular surface of the radial head displaced inferior to the capitellum. He was operated on 16 days after initial presentation, due to the patient not being available for surgery at an earlier time (Figure 7).

In the operating theatre, his radial head fragments were excised and the wrist, forearm, and elbow were screened in order to exclude an Essex-Lopresti injury. He then had an RHR and a lateral ulnar collateral ligament repair, which was avulsed from the humeral origin, and at the end of the procedure, he had a stable elbow across the entire range of movement. He was discharged on the same day, with rehabilitation commencing within a few days.

**Figure 8:**
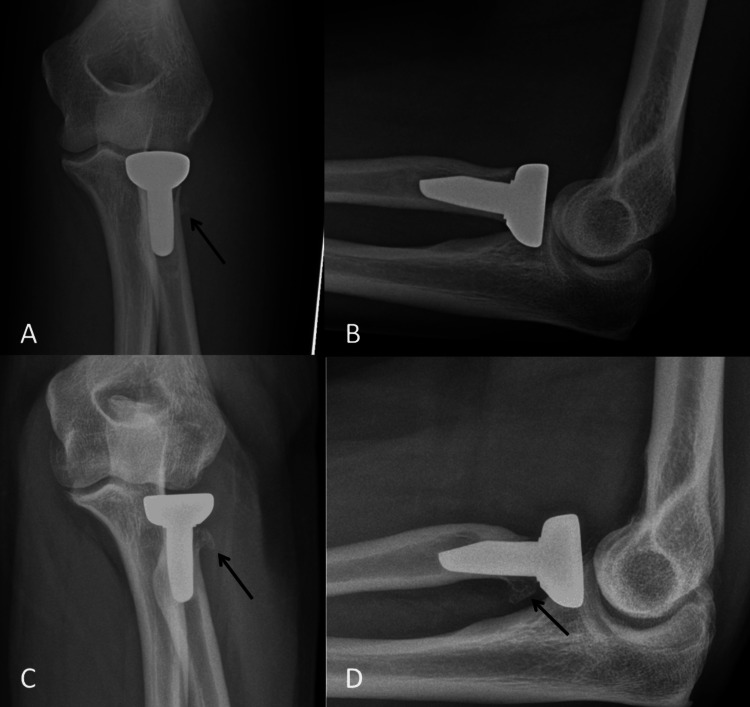
Post-operative radiographs taken at three weeks and six months after surgery. A: Antero-posterior view at three weeks after surgery demonstrating the subtle appearance of callus lateral to the level of the radial neck (black arrow). B: Lateral view showing a well-sized and well-fitting RHR. C: Antero-posterior view at six months after surgery showing mature callus at the radial neck (black arrow) and a degree of remodeling of the bone. D: Lateral view at six months after surgery showing the mature callus (black arrow) and a degree of loosening of the RHR. RHR, radial head replacement

He had his first post-operative radiograph three weeks after his surgery and it was noted that he had a callus over the lateral aspect of the implant/bone interface. Since then, there has been further osteolysis of the radial neck and the patient presents with mild elbow pain and a clicking sensation around the proximal radius. Repeat radiographs were taken six months after surgery and at that point, he had a normal white cell count and C-reactive protein. His range of movement at 24 months after surgery presents with full pronation and supination, although pronation past neutral is painful and he reports a clicking sensation. He can move from 20° to 120° in the flexion-extension arc. He has been offered a revision or excision of the RHR and we are awaiting his decision on this (Figure 8).

## Discussion

Radial head fractures were initially classified by Mason in 1954 into three categories and despite the fact that it has since been modified to an extent, this classification system is still in wider use [6]. Type 1 fractures may be treated non-surgically with early mobilization, type 2 fractures may be treated with or without surgery, depending on the presence of mechanical obstruction to rotation, and type 3 fractures are usually treated surgically, either with fixation of the fracture or with an RHR. Use of these implants continues to increase across the world, and National Joint Registry data suggest that the incidence of RHRs has increased threefold between 2012 and 2023, in England, Wales, and Northern Ireland [7].

In the context of the terrible triad of the elbow, there is evidence to suggest that RHR results in improved elbow stability compared to open reduction and internal fixation of these fractures and fewer post-operative complications [8,9].

When inserted appropriately, an RHR reproduces the biomechanical characteristics of the radio-capitellar joint and allows for the restoration of the stabilizing function of the radial head [10]. There are, however, several reported complications that may occur following an RHR.

These complications may be divided into general complications, implant design and fixation issues, and soft tissue-related complications.

General risks include prosthetic infection and posterior interosseous nerve injury during resection of the radial neck and relevant instrumentation, due to the nerve’s proximity to the surgical field [11]. In our practice, when we have to instrument the radial neck more distally than usual, we ensure that the posterior interosseous nerve is identified and protected throughout the procedure, while the forearm is pronated.

Implant-related complications have been reported across various studies. These include aseptic loosening, overstuffing of the radio-capitellar joint, incorrect sizing of the radial head implant, and disassembly in the case of modular implants [7]. Any of the aforementioned complications may affect the functional outcome enough to warrant revision surgery [12]. A meta-analysis demonstrated that out of more than 1000 patients undergoing RHR, there was a pooled rate of prosthesis excision or revision at 10% within an average follow-up period of 38 months [13]. This study also highlighted that cemented implants tend to have a lower incidence of removal/revision compared to uncemented stems.

The issue of overstuffing of the radio-capitellar joint after RHR is a significant one [14]. This may result in altered load distribution across the joint surface, accelerated wear of the capitellar cartilage, and subsequent osteoarthritis development [14]. Understuffing of the radio-capitellar joint, on the other hand, has been associated with ulno-humeral joint degenerative changes [15]. Moreover, incorrect replication of radial head dimensions and anatomy could lead toward either persistent instability or limitation of range of movement [16].

Soft tissue-related complications are predominantly associated with heterotopic ossification (HO) leading up to stiffness in the elbow after surgery. HO is the formation of ectopic new bone in juxta-articular soft tissues, and although it may occur in any joint after surgery or other trauma, it is prevalent in the elbow, especially in the context of fracture and dislocations of the elbow and in combined fractures of the radial head and the proximal ulna [17,18]. A systematic review reported that nearly half of prosthetic revisions and/or excisions of RHRs were attributed either directly or indirectly to HO formation. The callus formation in our series should not be attributed to HO, because in all four patients it presented very early in the post-operative period, between three and four weeks after surgery. Also, in at least two of our presented cases, radial neck fractures/fracture propagation were directly noted, one on a post-operative CT and one during the index operation. Finally, in our four patients, there was no other evidence of HO in the rest of the elbow joint at the time of initial radiographs.

Based on our case series, we suggest that early callus presenting at the implant-bone interface in RHRs may represent a fracture line that was either present but not identified at the time of index surgery or created during the implantation of a press-fit implant. We hypothesize that this may result in reduced stability of the implant as it is inserted and subsequently, in failure of osseointegration of the implant to the bone [10]. In such cases, loosening is highly likely, as it happened in our three patients who had standard stems implanted at index surgery. When early callus is noted after an RHR with a press-fit implant, such patients should be monitored very closely and not discharged early. Another important point that our case series demonstrates is that checking radiographs around four weeks after surgery is necessary for RHRs and may change post-operative management for these patients.

## Conclusions

RHR provides an excellent solution for non-reconstructible radial head fractures and offers restoration of the biomechanics of the elbow joint and comparable functional outcomes when matched against native radial heads. However, it does present with a specific set of post-operative complications, one of which is aseptic loosening.

Based on our presented cases in this paper, we suggest that when callus is observed soon after an RHR using a press-fit implant, it should raise suspicion that there may be fracture propagation in the metaphysis and at the very least, alert treating clinicians to the risk of early loosening or an inferior clinical outcome for that specific patient. Such patients should be followed up closely for the aforementioned complications and should not be discharged early.
